# Se Status Prediction by Food Intake as Compared to Circulating Biomarkers in a West Algerian Population

**DOI:** 10.3390/nu12123599

**Published:** 2020-11-24

**Authors:** Moussa Belhadj, Latifa Sarra Kazi Tani, Nouria Dennouni Medjati, Yahia Harek, Majda Dali Sahi, Qian Sun, Raban Heller, Ammaria Behar, Laurent Charlet, Lutz Schomburg

**Affiliations:** 1 Analytical Chemistry and Electrochemistry Laboratory, Abou Bekr Belkaid University of Tlemcen, BP 119, 13000 Tlemcen, Algeria; sarra.kazitani@univ-tlemcen.dz (L.S.K.T.); nouria.dennouni@univ-tlemcen.dz (N.D.M.); y_harek63@yahoo.fr (Y.H.); fdali13@yahoo.fr (M.D.S.); beharammaria@gmail.com (A.B.); 2 Institute of Earth Science, University of Grenoble-Alpes and CNRS, BP 53, CEDEX 9, 38041 Grenoble, France; laurent.charlet@univ-grenoble-alpes.fr; 3 Institute for Experimental Endocrinology, Charité-Universitätsmedizin Berlin, Corporate Member of Freie Universität Berlin, Humboldt-Universität zu Berlin, and Berlin Institute of Health, Augustenburger Platz 1, CVK, D-13353 Berlin, Germany; qian.sun@charite.de (Q.S.); raban.heller@charite.de (R.H.)

**Keywords:** selenium, monitoring, food intake, predictive model, selenoprotein P, glutathione peroxidase 3

## Abstract

Algeria is the largest country in Africa, located close to the Mediterranean coastal area, where nutrients consumption varies widely. Local data on selenium composition of foods are not available. We postulated a close correlation between selenium status predictions from food consumption analysis with a quantitative analysis of circulating biomarkers of selenium status. Population characteristics were recorded from 158 participants and dietary selenium intake was calculated by 24-h recall. The average total plasma selenium was 92.4 ± 18.5 µg/L and the mean of selenium intake was 62.7 µg/day. The selenoprotein P concentration was 5.5 ± 2.0 mg/L and glutathione peroxidase 3 activity was 247.3 ± 41.5 U/L. A direct comparison of the dietary-derived selenium status to the circulating selenium biomarkers showed no significant interrelation. Based on absolute intakes of meat, potato and eggs, a model was deduced that outperforms the intake composition-based prediction from all food components significantly (DeLong’s test, *p* = 0.029), yielding an area under the curve of 82%. Selenium status prediction from food intake remains a challenge. Imprecision of survey method or information on nutrient composition makes extrapolating selenium intake from food data providing incorrect insights into the nutritional status of a given population, and laboratory analyses are needed for reliable information.

## 1. Introduction

Selenium (Se) is an essential trace element for humans, mainly acquired through the daily diet [1]. The amount of Se in food items is variable, and it is hard to predict the Se content of a particular diet; its accumulation in plants depends on soil Se content and other soil parameters in a given area [2]. Dietary factors also determine the Se bioavailability; foods with high protein content (meat, fish, seafood) are characterised as better sources of Se, whereas high fat may impair bioavailability [3]. Most fruits and vegetables provide little Se because of their low content of protein and high content of water. The estimation of the Recommended Dietary Allowance (RDA) constitutes a considerable challenge, as the Se metabolism in human subjects is a complex, dynamic and a multifactorial process, depending on inflammation, genotype, sex, overall health status and other factors, and the different disease risks associated with Se deficiency do not provide a unique, population independent and universally accepted threshold for insufficient intake [4,5,6]. Even though the biosynthesis of certain selenoproteins is strictly dependent on a sufficiently high Se intake and shows low expression in Se deficiency, a Se deficit is not mirrored in a particular clinical phenotype [7].

This may be due to a hierarchical distribution of Se within the body and between the tissues and enzymes, as the essential selenoproteins appear to be preferentially supplied with the limiting trace element, even in times of Se deficiency [8,9]. Circulating and easily accessible selenoproteins have been established as protein biomarkers of Se status in subjects with marginal Se status, i.e., glutathione peroxidases and selenoprotein P (SELENOP), as they correlate almost linearly with Se intake [4,10,11]. However, with a sufficiently high Se supply, these protein biomarkers stabilize at serum Se concentrations of approximately 90 to 120 µg/L, with SELENOP covering the broadest range of Se intake [11,12,13]. Serum or plasma Se concentrations below 70 µg/L are considered to indicate Se deficiency [4,11,12]. High intakes of selenomethionine are mirrored not only in selenoprotein biosynthesis but also in an increased Se content of all proteins synthesized, where selenomethionine may replace regular methionine and, e.g., leading to considerable amounts of Se in other circulating proteins, e.g., albumin [14].

The RDA in the United States was estimated based on plasma glutathione peroxidase (GPX3) activity maximisation, and set at around 55 µg/day [15]. The nutrition societies of Germany, Austria and Switzerland (D-A-CH) have recommended intakes of 70 µg/day for men and 60 µg/day for women, at a rate of approximately 1 µg of Se/kg of body weight, an amount required for almost full SELENOP saturation [13]. Based on the occurrence of adverse effects, i.e., selenosis, the Tolerable Upper Intake Level (UL) for an adult is set at 400 μg/day [16,17]. When this intake level is surpassed over an extended time period, e.g., due to the consumption of misformulated Se-containing supplements, symptoms of selenosis may develop including loss of finger-and toenails, of scalp and body hair, muscle or joint pain and fatigue [18]. Conversely, insufficient Se intake and low Se status have been associated with increased risk for malignant, cardiovascular or infectious diseases [19,20,21], and poor survival chances in cancer [22], sepsis [23], severe injury [24] or COVID-19 [25,26]. Accordingly, it is of high importance for the medical systems in developing and developed countries to obtain reliable information on the Se status on a population-wide scale. Different measures are used, including dietary assessments and the quantification of total serum or plasma Se or the activity of glutathione peroxidases and SELENOP as circulating biomarkers of Se status [4,11,12,27].

The options in developing countries are limited, and an assessment via a 24-h food recall would enable a fast, cost-efficient and convenient way of identifying risk groups with severe Se deficiency. This study therefore aims to test whether a straightforward assessment of 24-h food recall yields valuable insights into Se status by comparing the results to laboratory biomarkers of Se status in a selected population of the largest country of Africa, i.e., in subjects residing in Western Algeria. Association between the food groups and their intake with plasma Se and SELENOP is determined, and the interrelation between the dietary assessment and the predicted values of Se intake with the analytically quantified circulating biomarkers of Se status is evaluated. Unfortunately, our hypothesis was not supported by the results and the data obtained verify the notion that Se status cannot reliably be estimated by a single 24-recall in combination with food nutritional composition tables. Obviously, the Se contents in the particular food items varied strongly and constituted no food item-specific characteristics that could be deduced from a general food data bank. The influence of the concentration and availability of Se in the soil of production [28] in combination with the knowledge of the geographical origin of the major food items consumed along with repeated food intake assessment appears to constitute the essential information for obtaining meaningful insights into the Se intake of a given population.

## 2. Materials and Methods

### 2.1. Human Samples

This cross-sectional observational study was performed on the general population of Western Algeria, i.e., in the Tlemcen department. One hundred and fifty-eight subjects were recruited from the medical analysis laboratory at the University Hospital of Tlemcen, Algeria, from January 2018 to March 2019. A detailed and precise questionnaire on anthropometric and socio-cultural parameters was conducted by a face-to-face interview, enabling the collection of data on personal characteristics (age, gender, blood pressure and diagnosis of hypertension, smoking habits, level of education, profession, average income, marital status) along with anthropometric data (body weight and height, yielding body mass index (BMI)). The study was carried out following the Algerian law (25/2006, Resolution N°. 387), it was approved by the Scientific Committee of the University of Tlemcen, as well as by the Ethics and Deontology Committee of the University of Tlemcen. All participants signed an informed consent before participation. To be eligible, volunteers needed to have no history of any cancer, or a chronic illness affecting their nutritional status. Subjects following a special diet or taking dietary supplements were also excluded from the study.

### 2.2. Dietary Intake Assessment

The amount and nature of each food item consumed during the last day was calculated in grams per day from a 24-h recall. Colour pictures of food samples with the weight indicated were provided to help participants make their choices as accurately as possible [29]. The quantities were converted into Se intakes using Ciqual (2017), a food nutritional composition table available online and free of charge [30]. Finally, all the answers were reviewed and completed if necessary.

To estimate the contribution of different dietary sources to daily Se intake, the foods were divided into categories: fish and seafood, legumes (included lentils, beans, peas and chickpeas), eggs, meat, milk and dairy products, bread, fresh fruits, cereals (included pasta, rice, bread, couscous and all dishes made from flour or semolina), vegetables (included raw and cooked vegetables) and potatoes. Although potatoes and bread are included in vegetables and cereal, respectively, they were considered as separate food groups because of their high consumption rate.

### 2.3. Selenium Status Assessment

Circulating biomarkers of Se status were assessed in plasma, essentially as described [10,31]. Blood samples were collected by venipuncture into 4 mL heparinised tubes, plasma and erythrocytes were separated by centrifugation at 1100× *g* (relative centrifugal force) using a Sigma 2-16P centrifuge for 15 min, and then they were frozen at −80 °C until the analyses were performed. Total Se concentration in plasma was determined by total reflection X-ray fluorescence (TXRF) analysis using a spiked gallium solution as standard and a benchtop TXRF analyser (S2 Picofox, Bruker nano GmbH, Berlin, Germany), and tested for accuracy by using a Seronorm serum standard (Sero AS, Billingstad, Norway) as described [32]. Plasma SELENOP was quantified by a validated commercial SELENOP-specific ELISA (selenOtest^TM^, selenOmed GmbH, Berlin, Germany) as previously described [33]. Enzymatic activity of plasma GPX3 was determined by a coupled enzymatic test, monitoring the consumption of NADPH at 340 nm [34].

### 2.4. Statistical Analysis

Normal distribution of values was assessed by the Shapiro-Wilk test. Non-parametric test methods were assessed to investigate location shifts between groups (Mann-Whitney U test, Kruskal-Wallis test). Categorical variables were evaluated using Fisher’s exact test. Relationship between parameters was tested by Spearman’s correlation analysis. As this was an exploratory post-hoc analysis, all *p*-values were to be interpreted descriptively, and no adjustment for multiple testing was adopted. Variable selection was performed via stepwise AIC selection [35,36]. Differences between ROC curves were assessed by the DeLong’s test for two correlated ROC curves [37]. All statistical tests used an α-level of 0.05. The results were considered as statistically significant when the *p*-value was less than 0.05, and differences are marked as follows: *p* < 0.05 (*), *p* < 0.01 (**) and *p* < 0.001 (***). All statistical calculations were performed with R version 4.0.2 [38], applying the packages “tidyr” [39], “dplyr” [40], and “pROC” [41]. Figures were created by using the package “ggplot2” [42].

## 3. Results

### 3.1. Characterisation of the Study Cohort

The characteristics of the subjects enrolled in this cross-sectional study were assessed by face-to-face interviews. One hundred fifty-eight subjects were recruited in total. The mean age was 49 (CI: 46–51) years, and the majority were female (83.7%). On average, participants were overweight, with a mean BMI of 26.8 (CI: 26.9–28.5) kg/m^2^, and a fraction of subjects were hypertensive, current smokers and in a stable marriage. The majority of samples indicated that the subjects were non-deficient in Se status, with a small fraction only (7.6%) exhibiting a plasma Se concentration below the consented threshold of deficiency, i.e., below 70 µg/L (median (IQR): 59.9 (21.8, 69.4) versus 93.7 (70.8, 143.2) µg/L, *p* < 0.001) (Table 1).

The groups were very similar, and neither the anthropometric nor the sociodemographic parameters tested indicated a significant difference between the groups of subjects classified as Se-deficient or Se-replete (Table 1). As the subjects were divided into two groups according to plasma Se concentrations, plasma SELENOP levels showed the expected difference between the groups.

### 3.2. Assessment of Se Intake via the Food Categories Using Reference Composition Data

The 24 h food recall data were used to quantify absolute food intakes per food category. Data were then converted into Se intakes by using the food composition information from the ANSES French Food Composition Table Ciqual 2017, and compared between the two groups of Se-deficient (plasma Se < 70 µg/L) and the Se-replete (plasma Se > 70 µg/L) subjects (Table 2).

The diet-specific comparison of the groups with replete or marginal Se status revealed no particular food item that turned out to be significantly associated with the different Se status. Even the calculated total Se intake in the groups was not different, when comparing the subjects with measured Se deficiency (plasma Se < 70 µg/L) as compared to those with higher plasma Se status.

### 3.3. Comparison of Intake-Deduced Se Status with Plasma Se Status Biomarkers

**Figure 1:**
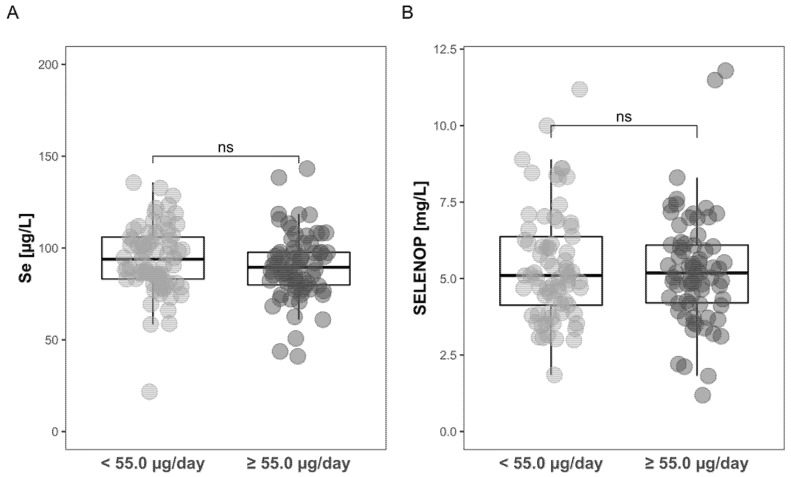
Comparison of calculated Se intakes per day as determined by the food recall method in combination with the food composition data in relation to the laboratory analysis of total Se and SELENOP concentrations measured in the plasma samples. No significant differences (“ns”) were detected between the two groups of different Se intakes (less or more than 55.0 µg Se/day) with respect to (**A**) total plasma Se, of (**B**) plasma SELENOP concentrations. Significance calculated by the Mann-Whitney U test, ns; *p* > 0.05.

The dietary food intakes were converted to daily Se intakes by the ANSES French Food Composition Table as highlighted above (Table 2). To test whether the results align with the expectation, i.e., providing an estimate on the resulting Se status, a direct comparison of the Se intake data with the measured Se status biomarkers was conducted. To this end, the subjects were divided according to their predicted Se intake, choosing the median Se intake as the threshold, i.e., whether daily intake was below or above 55 µg/day. The results indicate that the prediction of Se status based on the calculated daily Se intake and consumption pattern does not align with the Se status biomarkers measured, i.e., neither with total plasma Se nor with the protein SELENOP (Figure 1).

### 3.4. Interrelation of Plasma Se and SELENOP Concentrations in Se-Deficient vs. Se-Replete Subjects

**Figure 2:**
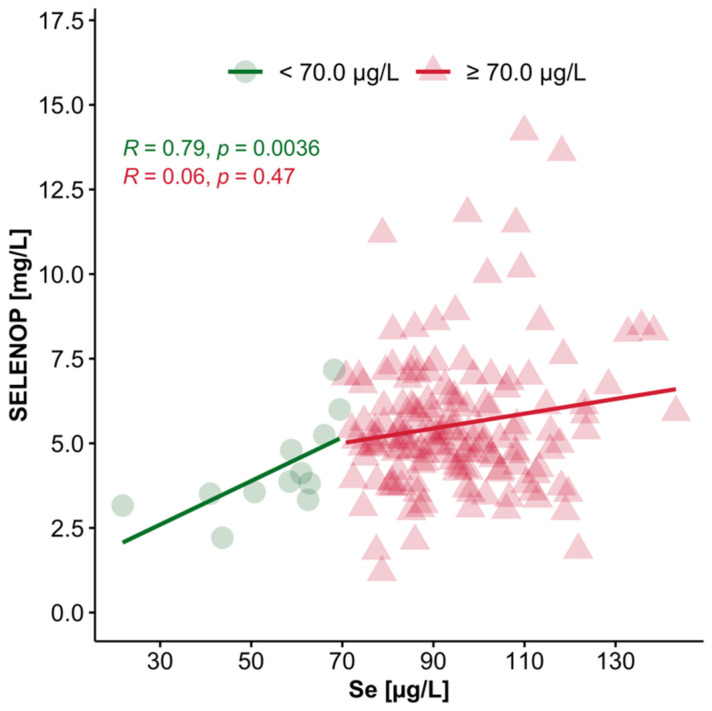
Correlation analysis of plasma Se with SELENOP concentrations. All of the available plasma samples (*n* = 134) of the patients enrolled were analysed for total plasma Se and SELENOP concentrations. The samples were separated into two groups based on total plasma Se deficiency into Se-deficient (<70 µg/L, green) and Se-replete (>70 µg/L, red). The biomarkers showed a significant and positive linear correlation (Spearman, *R* = 0.79, *p* = 0.0036) in the Se-deficient samples, whereas Se-replete subjects revealed a non-significant, positive correlation (Spearman, *R* = 0.06, *p* = 0.47).

The threshold for Se deficiency is generally considered to be a total serum or plasma Se concentration of 70 µg/L. Using this boundary, the total study cohort was divided into Se-deficient or Se-replete subjects. To analyse the interrelation of the two major Se status biomarkers, i.e., total plasma Se and SELENOP, this boundary was chosen to test the correlation of both biomarkers in the Se-deficient and Se-replete groups, respectively (Figure 2). The analysis indicates that there is a relatively tight and positive correlation between plasma Se and SELENOP concentrations, particularly in the Se-deficient subjects, with a weaker interaction in Se-replete subjects with plasma Se concentrations > 70 µg/L.

### 3.5. Interrelation of GPX3 Activity with Se Intake, Plasma Se and SELENOP

In a subset of the samples (*n* = 98), we were able to analyse the GPX3 activity; the other samples had to be excluded for reasons of either insufficient residual volume or compromised sample quality. The results were correlated with the concentrations of Se (*R* = 0.16, *p* = 0.12) and SELENOP (*R* = 0.04, *p* = 0.69). There was no significant correlation between the estimated Se intake and the GPX3 activity in the set of samples analysed (*R* = 0.01, *p* = 0.95).

### 3.6. Deducing a Model of Food Intake according to Food Categories Predicting Se Status

**Figure 3:**
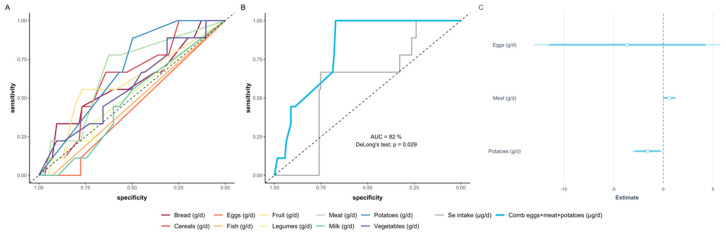
ROC-analysis of different food categories to differentiate between Se-deficient and Se-replete subjects. (**A**) Absolute intakes of several food categories (g/day) yielded similar results and poor predictive information. (**B**) The multiple regression model based on eggs, meat and potatoes intakes outperformed any other combination of variables via stepwise AIC selection. The final model (blue) based on these three parameters yielded a high AUC of 82%, and performed significantly better compared to the model based on the calculated Se intake (grey) from all categories deduced via the available composition data (DeLong’s test, *p* = 0.029). (**C**) The estimates of the final model are given alongside with the corresponding confidence intervals.

Finally, the data were used to model Se status from the data on food intake (amount and food categories) in relation to the measured biomarkers of Se status. The analyses indicate that information on the food categories eggs, meat and potatoes provided the most reliable match and outperformed any other combination of variables when compared via stepwise AIC selection (Figure 3).

## 4. Discussion

The essentiality of Se for human health is well established, and population-wide intake and status information is of high importance for the health care systems. However, the respective data are hard to obtain, and the best way to perform such analyses and how to predict Se status reliably has been intensively discussed [4,43,44]. In this study, we decided to compare nutritional Se intake prediction to laboratory analysis of Se status biomarkers in a North African population from Western Algeria. Our results indicate that the population on average consumes a wide variety of food items with some potentially Se-rich ingredients like sea food, meat, eggs and milk and leguminous plants. This impression is supported by the laboratory analyses of biomarkers of Se status including the most established parameters, i.e., total plasma Se, SELENOP and GPX3 [7,12,45]. Using the consented threshold for Se deficiency, i.e., serum or plasma Se concentrations below 70 µg/L, only a small fraction of less than 10% of subjects needed to be classified as insufficiently supplied with the essential trace element. However, there was no meaningful concordance when comparing the deduced Se status from the food intake patterns in combination with the food composition database with the measured biomarkers of Se status from the plasma samples. The most likely explanation for the observed mismatch between deduced values and measured concentrations lies in our assumption that using food composition data on Se contents of the different food categories would faithfully mirror the quality and Se content of the food items that have been consumed by the study participants. This assumption and strategy may yield accurate results for fat, carbohydrate or protein intakes, but unfortunately not for the trace element Se that presents itself again as difficult to grasp and predict, likely due to its complex geochemistry and uneven distribution [46,47,48].

Our laboratory analyses yielded average plasma Se and SELENOP concentrations in a range similar to what we determined in different European populations [31]. We did not observe a significant difference between men and women, which was in agreement with other independent studies on micronutrient status in Algeria [49,50], and also in agreement with other large population-wide studies in Europe [31], the US [51] or in Se-deficient or Se-replete areas of China [52]. Moreover, we did not observe a higher Se status in married as compared to single subjects, in contrast to a recent study [53].

The challenge of predicting Se intake from food frequency data is not new, and other attempts have similarly struggled with poor congruence, e.g., a respective study conducted in Finland [54]. The major reason for the inconclusiveness lies most likely in the varying Se content of a given food item, as it depends mostly on the area where it was produced and the respective soil quality and Se content [55,56]. In Algeria, most imported food groups are cereals (including wheat, meslin and corn) which cover more than 70% of its cereals needs. The cereals are grown in different regions of the world, mainly in America and Europe [57]. Similarly, milk, dairy products and legumes from different areas of the world contribute strongly to the Algerian nutrition [58]. The variation in the import origin of these products can be expected to have an impact on our analyses, as it causes strongly varying Se concentrations in the dietary items that formed the basis for our intake assessments and predictions [59].

On top of the variable international origin of the food items consumed in Algeria, local differences in Se content of the same nutrients are also known. Taking wheat as an example, a concentration range from as low as 21 µg/kg in Tiaret (western Algeria) to as high as 153 µg/kg in Khroub (eastern Algeria) has been reported in an Algerian study [60]. According to the Algerian Interprofessional office for Cereals, France is the main foreign supplier of cereals to Algeria [61]. French soil, as well as soils in other European countries, are rather poor sources of Se (with average Se contents as follows: France; 0.03 mg/kg, Finland; 0.08 mg/kg, Belgium; 0.11 mg/kg, Scotland; 0.17 mg/kg, Sweden; 0.30 mg/kg, and Norway; 0.63 mg/kg [62]) and are considered to be Se deficient [63]. Soils in other areas of the world, e.g., in the United States of America, can be richer sources of Se, with concentrations of up to 0.95 mg/kg [64].

Our data therefore highlight the need for laboratory-based analyses of Se status in a representative sample of a given population, and the challenge when trying to deduce Se status from nutritional intake data. Moreover, the data agree with prior studies reporting a relatively moderate Se status in Western Algeria, with a small fraction of subjects only displaying an insufficient daily intake. On the one hand, the globalization of the food industry and the associated transport of food items across the world pose environmental problems and contribute to climate change, but on the other hand these transports distribute the micronutrients more evenly across the populations and also into regions at risk of low supply. This noteworthy development clearly hinders food frequency-based predictions and complicates nutritional intake analyses, but it also contributes to better health by preventing severe deficiencies in areas where certain micronutrients are sparse. The complex origin of dietary Se in the average Algerian food serves as a most instructive example for this notion.

## 5. Conclusions

It appears impossible at present to correctly predict the average Se intake or resulting Se status of a given population from food intake information alone, at least as long as specific information on Se content of individual food items is not provided by the producers. Consequently, laboratory analyses of a representative sample of the population are needed to obtain the required information. To this end, different Se status biomarkers have been established and are available, and the results obtained usually agree reliably, especially in subjects with low Se status where insufficient intake causes low plasma Se levels and suppressed selenoprotein expression. Still, it would be helpful both for the health authorities and for the consumers alike to find specific information on the micronutrient contents on the commercial food items. This information should be provided at least for those nutrients that are imported in large amounts from remote areas of the world, in order to better justify transport, costs and virtual water economy [65], to better predict Se intakes and to more easily identify those subjects or groups at risk of insufficient Se intake. The relevance of this challenge is generally increasing, in view of the globally declining Se availability due to earth warming and climate change [66].

## Figures and Tables

**Table 1:** Comparison of subjects classified as Se-deficient ([Se] < 70 µg/L) or Se-replete.

	Se-Deficient	Se-Replete	Total	*p* Value
Total number (n)	12	146	158	
Age				0.728
median (IQR)	45 (30, 69)	46 (19, 90)	46 (19, 90)	
mean (95% CI)	47 (38, 56)	49 (46, 51)	49 (46, 51)	
BMI				0.208
median (IQR)	25.4 (19.5, 32.5)	27.2 (16.0, 43.2)	26.8 (16.0, 43.2)	
mean (95% CI)	25.8 (23.0, 28.5)	27.8 (27.0, 28.7)	27.7 (26.9, 28.5)	
Educational level				0.151
<high school	6 (54.5%)	68 (50.0%)	74 (50.3%)	
>high school	1 (9.1%)	43 (31.6%)	44 (29.9%)	
high school	4 (36.4%)	25 (18.4%)	29 (19.7%)	
Family income (k DZD) *				0.130
median (IQR)	28.0 (6.0, 200.0)	50.0 (8.0, 200.0)	47.5 (6.0, 200.0)	
mean (95% CI)	48.9 (13.7, 111.4)	54.7 (46.1, 63.4)	54.3 (45.6, 63.0)	
Gender				0.416
Female	9 (75.0%)	119 (84.4%)	128 (83.7%)	
Male	3 (25.0%)	22 (15.6%)	25 (16.3%)	
Hypertension				0.469
No	7 (63.6%)	103 (75.7%)	110 (74.8%)	
Yes	4 (36.4%)	33 (24.3%)	37 (25.2%)	
Marital status				0.626
Married	9 (81.8%)	120 (88.2%)	129 (87.8%)	
Single	2 (18.2%)	16 (11.8%)	18 (12.2%)	
Smoking				0.510
No	9 (81.8%)	95 (69.9%)	104 (70.7%)	
Yes	2 (18.2%)	41 (30.1%)	43 (29.3%)	
SELENOP (mg/L)				0.010
median (IQR)	3.84 (2.21, 7.17)	5.20 (1.19, 14.21)	5.11 (1.19, 14.21)	
mean (95% CI)	4.23 (3.37, 5.10)	5.56 (5.22, 5.89)	5.46 (5.14, 5.78)	

* A median income of around 50,000 Algerian Dinars (DZD)/month is equivalent to around 330€ or 390 US-$.

**Table 2:** Food intake-based analysis of absolute Se intake in Se-deficient and Se-replete subjects.

	Se-Deficient	Se-Replete	Total	*p*-Value
Bread (g/day)				0.307
mean (95% CI)	114 (45, 184)	154 (136, 171)	151 (134, 168)	
Cereals (g/day)				0.176
mean (95% CI)	152 (96, 208)	204 (184, 224)	201 (181, 220)	
Eggs (g/day)				0.344
mean (95% CI)	0.3 (−0.4, 1.1)	23.0 (13.4, 32.6)	21.5 (12.5, 30.5)	
Fish & Seafood (g/day)				0.382
mean (95% CI)	0.0 (0.0, 0.0)	9.1 (2.8, 15.4)	8.4 (2.6, 14.3)	
Fresh fruits (g/day)				0.263
mean (95% CI)	122.2 (24.0, 220.4)	80.4 (58.0, 102.8)	83.2 (61.5, 104.8)	
Legumes (g/day)				0.800
mean (95% CI)	53.4 (48.6, 155.3)	41.9 (24.1, 59.7)	42.6 (25.2, 60.1)	
Meat (g/day)				0.073
mean (95% CI)	67.8 (17.1, 118.4)	39.1 (28.3, 49.9)	41.0 (30.5, 51.6)	
Milk (g/day)				0.814
mean (95% CI)	56 (1.9, 109)	85 (57, 113)	83 (57, 110)	
Potatoes (g/day)				0.054
mean (95% CI)	22.2 (−5.7, 50.1)	85.9 (67.5, 104.4)	81.7 (64.2, 99.1)	
Vegetables (g/day)				0.443
mean (95% CI)	201.1 (84.3, 318.0)	253.4 (222.8, 284.0)	249.9 (220.6, 279.3)	
Calculated Se Intake (µg/day)				0.669
median (IQR)	77.0 (36.6, 84.8)	54.5 (17.8, 247.3)	55.2 (17.8, 247.3)	
mean (95% CI)	62.5 (48.6, 76.4)	62.7 (57.0, 68.3)	62.7 (57.4, 68.0)	
